# Infusion Reactions to Infliximab in Pediatric Patients with Inflammatory Bowel Disease

**DOI:** 10.3390/children11111366

**Published:** 2024-11-10

**Authors:** Rajmohan Dharmaraj, Tess Pei Lemon, Rasha Elmaoued, Ricardo Orlando Castillo, Razan Alkhouri

**Affiliations:** Division of Gastroenterology, Department of Pediatrics, University of New Mexico, Albuquerque, NM 87131, USA; lemont1@uthscsa.edu (T.P.L.); raelmaoued@salud.unm.edu (R.E.); ricastillo@salud.unm.edu (R.O.C.); ralkhouri@salud.unm.edu (R.A.)

**Keywords:** infiximab, infusion reaction, children, prevention, management

## Abstract

Infliximab (IFX) is a recombinant DNA-derived chimeric IgG monoclonal antibody protein that inhibits tumor necrosis factor alpha (TNF-α). IFX, like other agents derived from foreign proteins, can cause infusion reactions both during and after the infusion. The incidence of infusion reactions ranges between 0% and 15% in pediatric patients. The potential underlying mechanisms for these reactions may include anaphylaxis and anaphylactoid reactions, cytokine release syndrome, serum sickness-like reactions, and the development of antibodies against IFX. Several precautions can help reduce the risk of a new infusion reaction, such as a gradual increase in the infusion rate, scheduled infusions, and administering premedication or immunomodulators alongside IFX. Acute mild to moderate reactions often resolve spontaneously after a temporary cessation of the infusion or reduction in the infusion rate. Strategies like graded dose challenges and premedication can be utilized to prevent recurrence. In cases of severe reactions, desensitization or switching to an alternative biologic may be considered. This article aims to review the most recent guidelines for managing IFX-related infusion reactions in pediatric patients with inflammatory bowel disease (IBD), relying on the best available evidence.

## 1. Introduction

Infliximab (IFX) is a chimeric monoclonal antibody that targets tumor necrosis factor alpha (TNF-α), a proinflammatory cytokine that plays a key role in the pathogenesis of inflammatory bowel disease (IBD). IFX can be administered intravenously in the form of an infusion every 4–8 weeks after induction therapy, either in a hospital or outpatient setting. It is widely used in adult and pediatric IBD patients and has proven effective in inducing and maintaining remission. In IBD, the recommended intravenous (IV) dose of IFX for induction therapy and maintenance treatment is 5 mg/kg. The manufacturer has recommended that IFX be infused at a rate of at least 2 h [1]. It can be safely infused for a shorter duration without an increased risk of adverse infusion reactions [2].

Since IFX (Remicade, Janssen Biotech Inc., Horsham, PA, USA) was introduced for the first time in 1998, the Food and Drug Administration (FDA) approved eight biosimilars in the United States as of 2018. Biosimilars can be defined as biological products that are highly similar to an FDA-approved originator drug and have no clinically significant differences [3]. In pediatric IBD, the use of IFX biosimilars is gaining popularity. They are used for the initiation of therapy and, most recently, for switching from an originator product to a biosimilar. Biosimilars have been shown to be equally effective as originator medications for the induction and maintenance of remission [4,5,6,7]. The European Porto IBD Working Group of the European Society of Pediatric Gastroenterology, Hepatology, and Nutrition recently published a position paper stating that sufficient data exist for considering biosimilars as a safe and effective alternative to the originator drug [8].

However, the risk of adverse infusion reactions to IFX is well known. Most of these reactions are usually mild to moderate, and very few are severe, although the latter may still lead to the discontinuation of therapy. The exact etiology and pathogenesis of these reactions are often unclear, leading to conflicting findings on whether they are allergic or immune in nature. Hence, IFX infusion reactions are still poorly understood, and controlled and systematic data are lacking. Using the best evidence available, this article aims to review the most recent guidelines for managing the IFX-related infusion reactions among pediatric patients with IBD.

## 2. Materials and Methods

For this study, a bibliographic search was conducted through June 2024 in the PubMed, Embase, MedLine, and Cochrane databases. No language restrictions were applied. A language translation service was used to translate non-English studies into English. The search was performed with the assistance of a research librarian. No ethical approval was needed. Clinical trials, reviews, observational studies, and case reports were searched for. Three independent reviewers (RD, TL, and RA) evaluated and identified the relevant abstracts and titles from the search. Two authors (RD and RA) individually reviewed all the studies to check whether they included the reported pertinent data. The following were the inclusion criteria: children up to the age of 18 years with a confirmed diagnosis of IBD or a clinical subtype of IBD, ulcerative colitis (UC), Crohn’s disease (CD), receiving IFX induction or maintenance therapy, and having had the risk of infusion reaction evaluated. Articles that did not evaluate infusion reactions or those that focused on a different infusion therapy, such as vedolizumab, were excluded. Discussion and consultation among the coauthors resolved any of their disagreements.

## 3. Incidence

Infusion reactions to IFX are common in children with IBD. In a systematic review of pediatric IFX studies conducted in 2020, 498 patients had 347 standard and 3703 rapid infusions [2,9]. In the included four studies, the rate of infusion reactions reported per patient ranged between 0% and 15% for standard infusions and between 0% and 10% for rapid infusions. A retrospective study involving 796 patients with CD and UC analyzed a total of 5581 infusions [10]. The results indicated a higher rate of infusion reactions during induction infusions (14.3%) compared to maintenance infusions (6.8%) (*p* = 0.0135). Additionally, the overall incidence of infusion reactions per infusion was 2.0% for CD patients and 1.9% for UC patients, which was not statistically significant (*p* = 0.9224). The study found that women were more likely to experience infusion reactions than men (*p* < 0.0001). Furthermore, infusions administered in a patient’s home were associated with a lower rate of reactions compared to those given in an Ambulatory Infusion Suite, with rates of 1.6% versus 2.4%, respectively (*p* = 0.03). Infusions of IFX biosimilars were not associated with an increase in the incidence of infusion reactions for both standard and rapid rates in adults and children [11,12].

Immediate infusion reactions can occur within 24 h of starting an infusion, and most of the reactions occur within the first 2 h of infusion. Immediate reactions were reported in 5–23% of IBD patients [13,14,15,16,17]. In terms of severity, these reactions can range from mild to severe and include pruritus, erythema at the site of infusion, fever, chills, dyspnea, hypotension, and anaphylaxis. In contrast, delayed infusion reactions can occur between 24 h and 14 days after an infusion, and the majority of symptoms appear within 5–7 days. Delayed reactions were reported in 1–3% of IBD patients [18,19,20,21].

Severe infusion reactions can affect the ability to complete infusions, the duration of infusion, its effectiveness, and its long-term durability. Warnings and precautions regarding infusion reactions are now included in the prescribing instructions for all monoclonal therapeutic antibodies.

## 4. Factors and Mechanisms

Although exactly how IFX can cause infusion reactions is still unknown, several potential mechanisms have been suggested.

### 4.1. Anaphylaxis and Anaphylactoid Reactions

Anaphylaxis is a systemic acute reaction that occurs when mast cells release large amounts of histamine or other cytokines. This reaction is mediated by immunoglobulin E (type 1 IgE-mediated hypersensitivity reactions). IFX infusion reactions are often associated with symptoms that suggest an anaphylactic response. However, evidence of IgE mediation can be demonstrated only in a few cases, indicating that most anaphylactic-like reactions to IFX are not true type I hypersensitivity reactions but anaphylactoid reactions [22,23,24]. Sun Hwang and colleagues reported a case of anaphylaxis induced by infliximab, identifying an IgE-binding component and suggesting that the IgE-mediated response was a pathogenic mechanism [24]. The laboratory studies of this patient showed an elevated serum total IgE level of 688 kU/L, significantly above the upper limit of normal (120 kU/L). Additionally, IgE immunoblot analysis using the patient’s serum revealed a distinct band of 149 kDa that was not present in two control samples.

Anaphylactoid reaction is a systemic acute reaction that cannot be clinically differentiated from the anaphylactic response; however, it does not involve IgE. Such a reaction can be attributed to the direct activation and degranulation of mast cells by the drug, whether through IFX targeting membrane-bound TNF or by the release of anaphylatoxins (C3a, C5a) following complement activation. Anaphylactoid and anaphylactic reactions are serious and potentially fatal [25].

### 4.2. Cytokine Release Syndrome

The term “cytokine release” was initially coined to describe the rapid release of both pro- and anti-inflammatory cytokines and vasoactive substances by immune cells, following the treatment with antithymocyte globulin, anti-T-cell antibody muromonab-CD3 (OKT3), and rituximab [26,27]. Several possible mechanisms may be involved, including immune cell hyperactivation, complement-mediated lysis, direct apoptosis, and antibody-dependent cellular toxicity [28]. Similarly, immune cells expressing TNF, influenced by IFX, have been suggested to simultaneously release large amounts of cytokines [29,30]. This phenomenon is likely reflected in the paradoxical rise in serum TNF, which occurs immediately after the initial IFX administration [31].

Substantial overlaps are observed in the clinical symptoms of immediate infusion reactions caused primarily by the mentioned mechanisms, making it quite difficult to distinguish the exact immune or allergic nature of the response based solely on clinical evidence. However, some hints may be helpful. IgE-mediated responses require prior sensitization, and they should not occur during the first infusion. The presence of urticaria and wheezing may indicate a large release of histamine secondary to IgE mediation or mast cell degranulation. Alternatively, the presence of fever suggests that the infusion reaction is caused by cytokine release.

### 4.3. Serum Sickness-like Reactions

The serum sickness reaction (type III hypersensitivity reaction) is caused by the tissue deposition of antigen–antibody complexes that circulate within the blood. Laboratory evaluation usually shows signs of inflammation, including elevated ESR/CRP levels, the presence of immune complexes, including an increased percentage on C1q binding assay, and the activation of the classic pathway complement, including decreased C3 and C4 levels [32]. Serum sickness symptoms typically include a rash, fever, polyarthralgia, or polyarthritis, which appear 2–4 weeks after exposure. They resolve within a few weeks of discontinuing the agent responsible for it. Delayed infusion reactions to IFX can mimic the symptoms of serum sickness; however, they lack the characteristic laboratory findings [22]. These reactions, also called serum sickness-like reactions, rather than true serum sickness reactions, are due to the formation of human antichimeric antibodies or antibodies to IFX (ATI). ATI can cause various local and systemic inflammatory responses due to the fixation and activation of complements by antigen (IFX) antibody (ATI) immune complexes in blood vessels, the skin, and joint tissue.

### 4.4. Antibodies to Infliximab

Usually, ATI develops soon after starting treatment in most patients. Several studies have shown that the presence of ATI increases the risk of infusion reactions, and an increased risk exists with episodic or on-demand regimens, the resumption of IFX infusions after a prolonged drug-free interval, and in patients with high ATI titers [33,34].

In a retrospective cohort study conducted by Baert et al., a significant correlation was found between the concentration of ATI and the incidence of infusion reactions [35]. The study revealed that the median concentration of ATI was 20.1 µg/mL during the first infusion reaction, as opposed to 3.2 µg/mL in patients who did not experience an infusion reaction (*p* < 0.001). Concentrations of 8 µg/mL or higher were also observed to predict a higher risk of infusion reactions (*p* < 0.001).

Farrell et al. demonstrated that ATI-positive patients had a 40% incidence of infusion reactions compared to 4.7% in ATI-negative patients (*p* = 0.0001). Severe infusion reactions were noted in 28% vs. 0%, respectively (*p* = 0.0001) [36].

ATI is primarily linked to mild to moderate infusion reactions rather than severe reactions. Although ATI typically varies between 6% and 15%, it has been observed in 6–61% of patients with CD [36,37,38]. The wide range of ATI incidence can be attributed, in part, to the varying presence of risk factors for their development and the differences in the studied populations, such as treatment schedules, dosages, and comedications. Evidence suggests that combination therapy with 6-mercaptopurine, azathioprine, or methotrexate and corticosteroids can help reduce ATI formation [38,39].

It has also been shown that the carriage of HLA-DQA1*05 almost doubles the rate of ATI development, independent of immunomodulator use, for both IFX and adalimumab [40]. To minimize the risk of immunogenicity, pretreatment genetic testing for HLA-DQA1*05 may help personalize the choice of anti-TNF and the need for combination therapy with an immunomodulator.

## 5. Clinical Manifestations

Immediate infusion reactions to IFX can occur within 24 h of the beginning of an infusion, and most of the reactions occur within the first 2 h after infusion. Delayed infusion reactions occur between 24 h and 14 days following an infusion, and most symptoms appear within 5–7 days. The severity of symptoms can further be classified into mild, medium, or severe in both types of reactions, depending on the severity of the symptoms [41].

The most common clinical signs and symptoms of immediate reactions include a macular rash, which disappears in minutes or even hours, chest pain, dizziness, shortness of breath, headaches, hypotension or hypertension, nausea, sweating, and hyperthermia. Anaphylaxis can manifest as urticaria, laryngeal edema, and bronchospasm [42]. Immediate reactions are rarely anaphylactic (type 1 IgE-mediated hypersensitivity reactions) and, in most cases, are anaphylactoid in nature (nonallergic but with an immune etiology). Anaphylactic reactions often include dyspnea, chest tightness, hypotension, bronchospasm, and urticaria. To be classified as anaphylactic, an immediate reaction must have bronchospasm and urticaria. If neither of these symptoms is present, the reaction is likely anaphylactoid in nature. Premedication and a slow rate of infusion can be helpful in preventing anaphylactoid reactions [41]. Desensitization protocols can be used, or alternative biological agents can be considered in patients with true anaphylactic reactions [25].

Skin rashes, arthralgias, malaise, and myalgias are all symptoms of delayed infusion reactions. These reactions are analogous to mild serum sickness and may be classified as type III hypersensitivity reactions caused by immune complexes. The delayed reaction must be distinguished from similar states, including a viral concomitant syndrome and a lupus-like reaction, which occurs very rarely in TNF-α-blocking agents.

## 6. Primary Prevention

### 6.1. Infusion Protocol

The manufacturer of the IFX originator recommends that the initial (loading) infusion be given in a very controlled manner, starting with a small test dose and escalating the rate of infusion in a step-by-step manner until it reaches the target rate [1]. A standard 2-h infusion protocol is recommended for patients who can tolerate the initial infusions without complications (Table 1) [1]. The infusion can also be shortened to 60 min for patients who are tolerant of the 5 mg/kg infusions over 2 h [43]. The administration of 10 mg/kg over 60 min and 5 mg/kg over 30 min also appears to be safe [44,45].

Thus far, no controlled study seems to have validated the efficacy and safety of a gradual infusion schedule for preventing immediate reactions. However, this approach would appear prudent, as the cytokine release mechanism is the most common cause of these reactions.

### 6.2. Premedication

To prevent infusion reactions, premedications, which include acetaminophen, antihistamines, or corticosteroids, are routinely administered before the infusion. No conclusive evidence exists to prove that premedication can prevent infusion reactions in children with IBD (Table 2) [9,46,47,48,49,50,51].

In a comprehensive retrospective analysis of 1652 infusions in 243 children with IBD, 33 patients received premedication before the first infusion reaction, and 210 patients did not receive premedication until the development of the infusion reaction [46]. Infusion reactions were more common among patients who received premedication before the first reaction (12/33 versus 28/210, *p* < 0.01). The study did not find any benefit of using antihistamines, antipyretics, or corticosteroids before the first infusion reaction.

El-Matary et al. reviewed a total of 4120 rapid IFX infusions in children with IBD in a multicenter North American study [47]. Of those, 267 (59%) participants (2858 infusions) received one or more premedications that included corticosteroids (39 participants; 402 infusions), antihistamines (mainly diphenhydramine) (198 participants; 2293 infusions), and acetaminophen (260 participants; 2808 infusions). Although fewer infusion reactions with premedication were observed as a trend, the overall use of premedication was not significantly associated with infusion reactions’ occurrence compared with no use of premedication (adjusted relative risk (RR) = 0.61; 95% CI, 0.36–1.03; *p* = 0.06). However, the study showed a reduced risk of infusion reactions with antihistamine premedication (adjusted RR = 0.29; 95% CI, 0.14–0.64; *p* = 0.002) as compared to more frequent infusion reactions with the use of corticosteroids (adjusted RR = 8.42; 95% CI, 4.64–15.25; *p* < 0.0001).

Szymanska et al. studied the impact of premedication on 105 children who received a total of 1276 infusions [48]. Among the children, 55 received premedication (720 infusions), 44 were male, and the average age was 15 years old. The infusions included the IFX originator drug (Remicade) and two IFX biosimilars: Remisma and Flixabi. In terms of the incidence of infusion reactions, the study found no significant difference between the premedication+ and premedication− subgroups, with rates of 18.2% and 16.0%, respectively. In addition, the percentage of infusions followed by infusion reactions was not significantly different between the two subgroups (2.02% for premedication+ and 1.02% for premedication−). The odds ratio (OR) of developing an infusion reaction when using premedication was 0.34, and the difference in the infusion reaction ratio in premedication+ and premedication− patients was not statistically significant (95% confidence interval, 0.034–1.9). Of all the patients, 61.1% of severe infusion reactions (anaphylactic shock) were reported, regardless of premedication status. The study concluded that premedication, whether with steroids or antihistamines, did not reduce the incidence of the infusion reactions in pediatric patients with IBD receiving IFX.

Without evidence of a protective impact, the routine administration of premedication to patients remains questionable. The risk of side effects is unjustified, although these are low-risk agents. In contrast, premedication following a prolonged IFX-free interval may have a prophylactic impact, which has yet to be studied.

### 6.3. Infusion Schedule

The loading dose of IFX is administered in three doses at 0, 2, and 6 weeks, which is thought to be less immunogenic. Candon et al. found that in a retrospective study of 28 children with refractory luminal or fistulizing CD, three children (15.7%) developed ATI with a loading schedule at weeks 0, 2, and 6, while seven (77.7%) had antibodies with only one initial infusion [52]. Kugathasan et al. analyzed episodic IFX treatment over 30 months in 86 adult and pediatric patients receiving 304 infusions and demonstrated higher ATI formation and high rates of severe systemic reactions in patients receiving a distant second infusion (≥20 weeks from the first infusion) [33].

To prevent ATI formation and infusion reactions, maintenance treatment is recommended every 8 weeks instead of the on-demand option. Hanauer et al. analyzed the effects of different treatment regimens on ATI formation using data from the ACCENT I trial in 573 patients with CD [18]. The patients received 5 mg/kg IFX (week 0) and then were randomly assigned to blinded infusions of a placebo at weeks 2 and 6 and every 8 weeks thereafter until week 46 (group I), 5 mg/kg IFX at weeks 2 and 6, and every 8 weeks thereafter until week 46 (group II), or 5 mg/kg IFX at weeks 2 and 6, followed by 10 mg/kg thereafter (group III). At week 14 or later, patients losing a response were switched over to episodic IFX treatment, in which IFX was increased by 5 mg/kg. The authors observed a reduced antibody formation and greater clinical benefit with an induction regimen followed by maintenance treatment compared with a single dose followed by episodic retreatment in CD patients treated with IFX.

### 6.4. Immunomodulators

The risk of infusion reactions appears to be lower when IFX is coadministered with thiopurines in the adult population [18,35,53]. Such combination therapy can also improve the efficacy of IFX and decrease its immunogenicity [18]. Methotrexate was also shown to be effective in preventing infusion reactions among patients who received an on-demand regimen of IFX [53]. However, no definitive evidence was observed to show that the coadministration of immunomodulators with IFX can prevent infusion reactions in children with IBD (Table 3) [9,47,49,50,54]. While immunomodulators may be efficacious in preventing immediate reactions during an infusion, they should still be carefully considered in terms of safety concerns, such as the risk of infection and lymphoma [55].

## 7. Management

**Figure 1:**
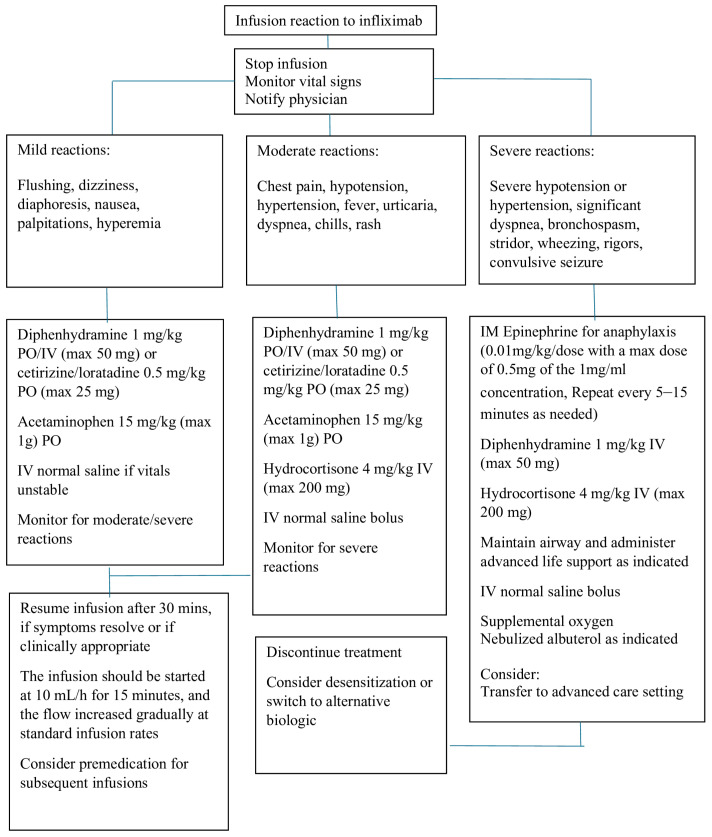
Algorithm for management of infliximab infusion reactions.

Most patients with an acute reaction to infusions can be successfully managed, and the treatment can continue without discontinuing the infusion. The symptoms can be managed by decreasing the infusion rate and using IV fluids and medications, including antihistamines, acetaminophen, and steroids. In the case of a true anaphylactic response, the infusion should be stopped, and epinephrine should be administered, followed by IV steroids. Patients can either be treated with adalimumab, which is fully human TNF-alpha, or desensitized. A suggested algorithm for managing immediate infusion reactions is outlined in Figure 1.

Mild to moderate infusion reactions can be treated by slowing down the rate of infusion, while severe reactions require emergency action [37,41,56,57,58,59,60,61], such as stopping the infusion immediately and providing appropriate medical treatment and supportive care. Medications, including corticosteroids, IV antihistamines, bronchodilators, oxygen, and vasopressors, should be available when IFX is infused. Infusion room nurses and physicians should be well prepared to manage severe infusion reactions.

IgE-mediated responses can manifest very quickly and with a severe reaction, whereas some non-IgE-mediated reactions are also severe and may mimic anaphylaxis. Clinicians may be unable to distinguish between IgE and non-IgE reactions. In the case of IgE-mediated anaphylaxis, epinephrine (EpiPen) should be immediately administered with IV steroids and antihistamines. A joint task force of professional associations in allergy, asthma, and immunology has recently published guidelines for anaphylaxis diagnosis and treatment [42]. These guidelines recommend epinephrine as the first-line therapy for life-threatening symptoms, such as stridor, respiratory distress, wheezing, hypotension, arrhythmia, seizures, or shock.

Determining the best treatment for delayed infusion reactions is difficult due to a lack of data and the rarity of these reactions. Antihistamine is often prescribed for relief from the symptoms of pruritus. Acetaminophen can be used to relieve low-grade fevers and arthritis. Oral corticosteroids are often prescribed for patients with high fevers, severe arthritis/arthralgia, or widespread rash/pruritus. IV corticosteroids may be used in acutely ill patients. The treatment and regimen are prescribed based on the physician’s judgment, both in terms of dosage and duration. If the delayed infusion reaction is mild and quickly resolves, retreatment using IFX may be recommended using a prophylactic regimen similar to that used in the treatment of acute reactions. Some authors suggest increasing the IFX doses or shortening the intervals between infusions to increase the IFX concentrations and reduce the immune complex formation [62].

## 8. Secondary Prevention

Whether IFX can continue to be used after an infusion reaction is often questioned. This depends on the severity of the infusion reaction, the effectiveness of treatment, and the availability of treatment alternatives. The benefits and risks of continuing IFX should be carefully considered after a severe infusion reaction. IFX is not usually discontinued following a mild or moderate reaction. Various precautions can be taken to reduce the risk of a new infusion reaction, including adjustments to the dosage and infusion schedule, premedication, and desensitization.

### 8.1. Graded Challenge

If a patient has experienced an acute infusion reaction to IFX and needs to be treated again, beginning with a small test dose is recommended, followed by gradually increasing the infusion rate until the full target rate or the maximum tolerated rate is achieved [22]. If most immediate infusion reactions are caused by cytokine release, then any reactions from the smaller test doses are believed to be milder and easier to manage. Although this assumption has not been formally validated through controlled studies, in practice, most infusion centers widely accept graded dose challenges.

### 8.2. Premedication

The preemptive use of antihistamines, antipyretics, and/or corticosteroids is often employed to prevent the recurrence of infusion reactions. However, no solid evidence exists to prove the prophylactic effect of premedication, and no widespread consensus exists on the specific indications and criteria, choice of medications, dosage, or administration method.

A study conducted by Jacobstein et al. examined 243 children with IBD who received IFX infusions [46]. Among the 28 patients who experienced an infusion reaction without premedication, 10 (36%) received retreatment with premedication, 12 continued without premedication, and 6 did not have any further infusions recorded. Of the 10 patients who started receiving premedication, 2 had a subsequent infusion reaction, whereas 6 out of 12 who did not receive premedication had another reaction (*p* = 0.15). The study discovered that treating children who had previously experienced infusion reactions without any premedication significantly increased their chances of experiencing infusion reactions (50%). When children were premedicated with antihistamines, antipyretics, or corticosteroids, the likelihood of recurrent infusion reactions appeared to be lower. However, Colombel et al. observed that premedication did not decrease the risk of recurrent infusion reactions in the adult population [63].

Although no solid evidence proving the preventive effect of premedication exists, it is noteworthy that providing premedication before an infusion can potentially decrease the severity of an infusion reaction and increase the chances of completing the infusion. Patients with a history of moderate infusion reactions may benefit from premedication, although many patients may need to be treated for one to receive the benefit. Treatment should be discontinued in cases of severe and potentially life-threatening acute generalized infusion reactions.

### 8.3. Desensitization

Desensitization is an option if IFX must be continued after a severe infusion reaction. Desensitization should be avoided if alternative treatment options are available. The desensitization procedure should be performed with safety precautions taken and by an experienced physician, with emergency equipment available.

The process of desensitization to a specific medication was first described for IgE-mediated anaphylactic reactions. It involves gradually administering the offending drug, starting with very low doses (at dilutions of 1:1000 and 1:100), and then slowly increasing the dose until the target dose can be tolerated clinically. Continuous low-level exposure to the antigen appears to make tissue mast cells and possibly circulating basophils less responsive to the offending drug while still responding to other stimuli [23].

The information on the desensitization to IFX is based mainly on small series studies and case reports [64,65,66]. In these studies, the rate of breakthrough reactions was reported to be as high as 29%, which is similar to that of reaction recrudescence observed without desensitization [64]. However, these reactions were generally milder and often allowed for the successful continuation of the infusion.

### 8.4. Biologic Switching

Switching biologic therapy is the replacement of one biologic with another within the same class or outside the class of the previous biologic. Switching can be considered if all the measures to avoid infusion reactions fail and the decision is made to stop IFX infusions. As described in the literature, patients can be switched from IFX to adalimumab without complications after a severe infusion reaction [67,68,69]. In a 4 wk open-label trial evaluating the efficacy of subcutaneous adalimumab as an induction therapy in patients with UC who had an attenuated response or had become intolerant (n = 4) to IFX therapy, none of the patients experienced intolerance to adalimumab that led to the discontinuation of treatment [68]. Another study evaluated the short- and long-term outcomes of adalimumab in 30 patients with UC who had previously been treated with IFX, including 12 patients who were intolerant to IFX [69]. The findings indicated that adalimumab was well tolerated and led to a durable clinical response in many patients with otherwise medically refractory UC. The study concluded that patients with UC who are intolerant of IFX may benefit from switching to adalimumab.

## 9. Conclusions

Infusion reactions to IFX are common in children with IBD. Although no solid evidence proving the preventive effect of premedication exists, patients with a history of moderate infusion reactions may benefit from premedication. Co-administration of immunomodulators with IFX may help prevent immediate reactions; however, this approach should be weighed against the increased risks of infections and malignancies. Desensitization or switching to an alternative biologic therapy can be considered in patients with severe reactions. There is still a lack of systematic and controlled data regarding the risk, prevention, and management of infusion reactions to IFX in the pediatric population. Therefore, there is an urgent need for well-designed controlled trials to investigate the effectiveness of the proposed preventive and management strategies.

## Figures and Tables

**Table 1:** Infliximab Rate Protocols.

Standard Infusion Rates		Rapid Infusion Rates	
250 mL, 120 min (Doses < 1000 mg)	500 mL, 120 min (Doses ≥ 1000 mg)	250 mL, 60 min (Doses < 1000 mg)	500 mL, 60 min (Doses ≥ 1000 mg)
10 mL/h × 15 min	10 mL/h × 15 min	20 mL/h × 8 min	50 mL/h × 8 min
20 mL/h × 15 min	20 mL/h × 15 min	40 mL/h × 8 min	100 mL/h × 8 min
40 mL/h × 15 min	40 mL/h × 15 min	80 mL/h × 8 min	350 mL/h × 8 min
80 mL/h × 15 min	80 mL/h × 15 min	160 mL/h × 8 min	500 mL/h × 8 min
150 mL/h × 30 min	150 mL/h × 30 min	300 mL/h × 15 min	750 mL/h until completed
250 mL/h until completed (approximately 30 min)	250 mL/h until completed (approximately 90 min)	550 mL/h until completed	

**Table 2:** Summary of pediatric studies describing the use of premedication and infusion reactions.

Reference	Study Type	Cohort (n)	Premedication Agents	% IR with Premedication	% IR Without Premedication	*p*-Value
Van Wassenaer et al. [9]	Case-Control study	226	Corticosteroids	14.3	17.0	0.58 (NS)
Jacobstein et al. [46]	Retrospective review	243	Antipyretic, antihistamine, corticosteroid	36	13	<0.01 (S)
El-Matary et al. [47]	Retrospective review	453	Corticosteroids, antihistamines, acetaminophen			0.06 (NS)
Szymanska et al. [48]	Case-Control study	105	Corticosteroids (corhydrone/hydrocortisone)	18.2	16	>0.05 (NS)
Zeng-Wang et al. [49]	Retrospective review	185	Methylprednisolone	11.8	13.2	0.727 (NS)
Rozette et al. [50]	Prospective and retrospective study	116	Acetaminophen, cetirizine, diphenhydramine, loratadine, methylprednisolone			>0.05 (NS)
Lahdenne et al. [51]	Prospective observational and retrospective study	124	Acetaminophen, cetirizine	12.5	8.3	0.364 (NS)

IR, infusion reaction; NS, not significant; S, significant.

**Table 3:** Summary of pediatric studies describing concomitant immunomodulator medications and infusion reactions.

Reference	Study Type	Cohort (n)	Number (%) of Patients with Immunomodulators	% IR with Immunomodulators	% IR without Immunomodulators	*p*-Value
van Wassenaer et al. [9]	Case-Control study	226	74/91 in the premedication+ group (AZA 58, MTX 20) 96/135 in the premedication—group (AZA 76, MTX 9)	NA	NA	0.39 (NS)
El-Matary et al. [47]	Retrospective review	453	165 (36.4) AZA 55 MTX 101	RR = 0.31 (95% CI, 0.14–0.67)		0.003 (S)
Zeng-Wang et al. [49]	Retrospective review	185	123 (66.5) AZA 42 (22.7) MTX 15 (8.1)	16.7	38	0.048 (S)
Rozette et al. [50]	Prospective and retrospective study	116	Retrospective arm 15 (30) Prospective arm 50 (76) AZA 27 (41) MTX 18 (27)	NA	NA	>0.05 (NS)
Clare et al. [54]	Prospective study	144	107 (74.3) AZA 82 (56.9) MTX 20 (13.9)	NA	NA	0.69 (NS)

AZA, azathioprine; MTX, methotrexate; IR, infusion reaction; RR, relative risk; NS, not significant; S, significant; NA, data not available.
